# Electronic conduction in La-based perovskite-type oxides

**DOI:** 10.1088/1468-6996/16/2/026001

**Published:** 2015-03-10

**Authors:** Hisashi Kozuka, Kazushige Ohbayashi, Kunihito Koumoto

**Affiliations:** 1 NGK Spark Plug Co., Ltd, 2808, Iwasaki, Komaki-shi, Aichi 485-8510, Japan; 2 Department of Applied Chemistry, Graduate School of Engineering, Nagoya University, Furo-cho Chikusa-ku, Nagoya 464-8603, Japan

**Keywords:** electronic conduction, electrical conductivity, carrier concentration, carrier mobility, effective mass, relaxation time

## Abstract

A systematic study of La-based perovskite-type oxides from the viewpoint of their electronic conduction properties was performed. LaCo_0.5_Ni_0.5_O_3±*δ*_ was found to be a promising candidate as a replacement for standard metals used in oxide electrodes and wiring that are operated at temperatures up to 1173 K in air because of its high electrical conductivity and stability at high temperatures. LaCo_0.5_Ni_0.5_O_3±*δ*_ exhibits a high conductivity of 1.9 × 10^3^ S cm^−1^ at room temperature (R.T.) because of a high carrier concentration *n* of 2.2 × 10^22^ cm^−3^ and a small effective mass *m*∗ of 0.10 m_e_. Notably, LaCo_0.5_Ni_0.5_O_3±*δ*_ exhibits this high electrical conductivity from R.T. to 1173 K, and little change in the oxygen content occurs under these conditions. LaCo_0.5_Ni_0.5_O_3±*δ*_ is the most suitable for the fabrication of oxide electrodes and wiring, though La_1−*x*_Sr_*x*_CoO_3±*δ*_ and La_1−*x*_Sr_*x*_MnO_3±*δ*_ also exhibit high electronic conductivity at R.T., with maximum electrical conductivities of 4.4 × 10^3^ S cm^−1^ for La_0.5_Sr_0.5_CoO_3±*δ*_ and 1.5 × 10^3^ S cm^−1^ for La_0.6_Sr_0.4_MnO_3±*δ*_ because oxygen release occurs in La_1−*x*_Sr_*x*_CoO_3±*δ*_ as elevating temperature and the electrical conductivity of La_0.6_Sr_0.4_MnO_3±*δ*_ slightly decreases at temperatures above 400 K.

## 1. Introduction

This review focuses mainly on electronic conduction in La-based perovskite-type oxides to replace metal electrodes and wiring by electronic conductive oxides in ceramic-based products. Both high electrical conductivity and high stability at high temperatures in air are required of oxide electrodes and wiring in order to apply them to various ceramic products, though there are few reports for such oxides. Highly conductive and stable oxides from R.T. to about 1150 K in air are reviewed in this paper.

Electronic conduction is an important property in perovskite-type oxides. High-temperature superconductivity is a representative area of research related to electronic conduction. Since the discovery of the first high-temperature superconductor [1], the physical properties of many materials have been investigated at low temperatures [2–5] to gain a greater understanding of their essential properties. On the other hand, electronic conduction above R.T. is important for products used in daily life because it contributes to propagation of the electrical signals.

Many ceramic-based products, such as multilayer ceramic capacitors (MLCCs), low-temperature co-fired ceramics (LTCC), ceramic integrated circuit (IC) packages, negative temperature coefficient (NTC) thermistors and spark plugs, are composed of a combination of oxides and metals. The oxides provide the functionality of these products, whereas the metals, which are fine electron conductors, propagate the electrical signals. Fabricating these products is difficult because the physical properties of the base oxides and metals are very different. The quality of commercially manufactured products is achieved by designing production processes that account for the differences in synthesis temperatures, synthesis atmospheres, coefficients of thermal expansion and sinter shrinkage. However, the intrinsic performance of the base oxides may be suppressed because the optimal synthesis conditions for the base oxides are not necessarily consistent with the production process. Thus, in terms of matching base oxides with electrodes, conductive oxides should be more effective than metals as electrodes. If the metals used to form the electrodes for ceramic products can be replaced by conductive oxides, innovations in the ceramics industry will be realized. Moreover, oxide electrodes that are stable in air at high temperatures are expected to be used more broadly. To replace the metal electrodes and wiring in various ceramic products by oxides, oxides with high electrical conductivity of ≥ 1000 S cm^−1^ and high stability from R.T. to 1173 K in air are required, though such oxides have not attracted attention in an academic field. Therefore, in the present study, oxides with the potential to replace metal electrodes in air in the temperature range from R.T. to 1173 K were fabricated, and their physical properties above R.T. were investigated.

All the samples described in this study were synthesized using a conventional solid-state technique in air. Refer to previous reports for details [6–9]. Materials synthesized using this method are believed to be thermodynamically stable up to their synthesis temperatures. Similarly, the physical properties of the materials were evaluated in air. The relative densities of the specimens were ≥ 94% except for SrMnO_3_ and La_0.1_Sr_0.9_MnO_3_. The relative densities of SrMnO_3_ and La_0.1_Sr_0.9_MnO_3_ were ≥ 90%. As an example, the scanning electron microscopy images of La_0.6_Sr_0.4_MnO_3_ are shown in our previous report [9]. There were small pores of about 1 *μ*m in size, though the relative density exceeded 98%. The grain size was ≤ 10 *μ*m.

## 2. Selection of oxide systems

RuO_2_, SrRuO_3_, IrO_2_, LaNiO_3_, ReO_3_ and NbO are well known as fine electron conductors [10–15]. However, RuO_2_, SrRuO_3_, IrO_2_ and ReO_3_, which are used as thick-film resistors, are not suitable as practical replacements for metal electrodes and wiring because they contain expensive rare metal elements as main components. The usefulness of LaNiO_3_, a fine perovskite-type oxide electron conductor, is limited by its high-temperature stability (only to 973 K). NbO is also thermodynamically unstable at high temperatures in air. Consequently, LaCoO_3_- and LaMnO_3_-based perovskite-type oxides were selected as having potential for practical industrial use, given that (і) they do not contain any expensive rare metals, (іі) they are not environmentally hazardous and (ііі) they are stable up to 1173 K in air.

On the basis of its ionic radius and the number of 4f electrons, La was selected as the A-site element for perovskite-type oxides. In the perovskite-type structure, the tolerance factor and symmetry of the structure increase as the ionic radius at the A-site increases. As a result, La-based perovskite-type oxides tend to form a cubic or a rhombohedral phase, which is advantageous for electronic conduction because the ionic radius of La is the largest among the rare-earth elements. In contrast, an orthorhombic or tetragonal phase, which is disadvantageous for electronic conduction, is stabilized when the ionic radius at the A-site is smaller. Table 1 lists the electrical conductivities of RE_0.5_Sr_0.5_CoO_3_ complexes (RE = La, Pr, Sm, Gd and Tb) at R.T. The electrical conductivity is observed to increase as the ionic radius at the A-site increases [16].

**Table 1.** The electrical conductivity of RE_0.5_Sr_0.5_CoO_3_ at R.T.

RE	Ion radius (Å)^a^ [16]	Conductivity (S cm^−1^)
La^3+^	1.23	4.4 × 10^3^
Pr^3+^	1.14	3.5 × 10^3^
Sm^3+^	1.06	2.8 × 10^3^
Gd^3+^	1.04	3.2 × 10^2^
Tb^3+^	1.00	2.5 × 10^2^

a The coordination number is 12.

In addition, the fact that La^3+^ has no 4f electrons is also beneficial for electronic conduction because 4f electrons clearly contribute to the density of states (DOS) around the Fermi level, and this DOS degenerates strongly (i.e. the bandwidth is narrow and steep). In other words, the electrical conductivity decreases and the effective mass increases due to the presence of 4f electrons. It should be noted that Y^3+^ is not beneficial for electronic conduction because its ionic radius is small, although it also has no 4f electrons. For these reasons, La^3+^ was selected as the most suitable A-site ion for the fabrication of conducting oxides.

## 3. Effect of doping LaCoO_3_ with alkaline earth metals [6]

### 3.1. Characteristics of LaCoO_3_

Alkaline earth (AE)-doped LaCoO_3_ is an attractive material because it has one of the highest electrical conductivities among the oxides [17, 18]. As a result, AE-doped LaCoO_3_ has potential applications in nonvolatile memories [19], thermoelectric materials [17, 20, 21], solid oxide fuel cell (SOFC) cathodes [22, 23] and oxygen-permeable membranes [24, 25]. The metal–insulator transition is known to occur when LaCoO_3_ is doped with Sr [26]. However, the origin of the high electrical conductivity has not yet been clarified. Therefore, to verify the AE-doping effect, the systematic doping of perovskite-type oxides at the La-site using three AE elements (Ca, Sr and Ba) was investigated. Generally, the carrier mobility of perovskite-type oxides is believed to increase with the M–O–M bond angle (M is the B-site element) because the large overlap and strong interactions between the M(3*d*)–O(2*p*) orbitals lead to an increase in the relaxation time [27]. Based on this consideration, the alteration of the crystal structure due to the connection of the CoO_6_ octahedra through shared vertex oxygen ions, which occurs as a result of the employed synthetic method, is assumed to be one of the driving forces in this system. Therefore, the relation between the electrical properties and the crystal structure of LaCoO_3_ doped with three types of alkaline earth elements with differing ionic radii was evaluated in the range of 0 ≤ *x* ≤ 0.40 (*x* = AE concentration).

### 3.2. Crystal structure of AE-doped LaCoO_3_

**Figure 1.**
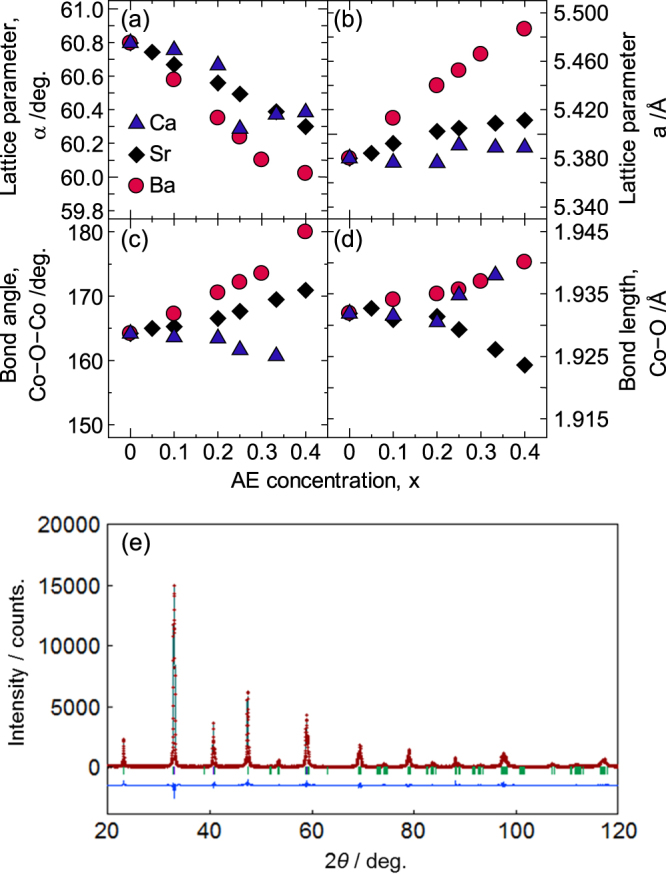
Lattice parameters of La_1−*x*_AE_*x*_CoO_3_ based on a rhombohedral unit cell: (a) lattice angle, (b) lattice length, (c) Co−O−Co bond angle and (d) Co−O bond length. Reproduced from [6] by permission of The Royal Society of Chemistry. Figure 1(e) XRD pattern for La_0.6_Sr_0.4_CoO_3_.

The perovskite-type structure of the doped LaCoO_3_ systems was confirmed via powder x-ray diffraction (XRD) analysis. The lattice parameters of La_1−*x*_AE_*x*_CoO_3_ and XRD pattern of La_0.6_Sr_0.4_CoO_3_ are shown in figure 1. Powder XRD patterns were measured with CuKa radiation at R.T. (RIGAKU, RINT TTR-III, 20 ≤ 2*θ* ≤ 120, step scan 0.02, 50 kV–300 mA). The lattice parameters were refined by means of Rietveld analysis using the RIETAN2000 code with the powder XRD patterns. The result of the Rietveld analysis for La_0.6_Sr_0.4_CoO_3_ (*R*_*wp*_ = 10.63%, *R*_*p*_ = 7.51%, *S* = 1.43) is shown in figure 1(e) as an example. The XRD patterns were shifted systematically with the average ionic radius at the A-site (i.e. the ionic radius of the AE element and the AE concentration). However, the patterns for La_1−*x*_Ca_*x*_CoO_3±*δ*_ became anomalous with the increasing Ca content (*x* ≥ 0.25). The values for the lattice length (*a*), the lattice angle (*α*), the Co–O–Co bond angle and the Co–O bond length of each of the doped LaCoO_3_ systems refined via Rietveld analysis using the rhombohedral *R-*3*c* space group are shown in figures 1(a) and (b) [28–31]. The different behaviors of *a* and *α* as a function of the AE concentration for Sr, Ba and Ca are reflected in the XRD patterns. The *a* and *α* values for Sr and Ba varied depending on the AE concentration and the ionic radius of the AE element, while there was an anomaly at approximately *x* = 0.25 for Ca. In addition, the Co–O–Co bond angle approached 180° when the average ionic radius at the A-site increased, as shown in figure 1(c). This result indicated that the conduction path, or the connection between the CoO_6_ octahedra, approached 180° when the average ionic radius at the A-site increased. In contrast, the Co–O bond length did not vary with the AE element, as shown in figure 1(d). Only the Co–O bond length for Sr decreased as a function of the AE concentration for the condition wherein *x* ≥ 0.25. Note that the values for the Co–O bond length and Co–O–Co bond angle in La_1−*x*_Sr_*x*_CoO_3±*δ*_ determined in the present study are not consistent with previous reports [32, 33] because of the difference in the oxygen content and the alteration of the CoO_6_ octahedra, which are a direct result of the synthetic method and the reaction conditions employed.

### 3.3. Electrical properties of AE-doped LaCoO_3_

**Figure 2.**
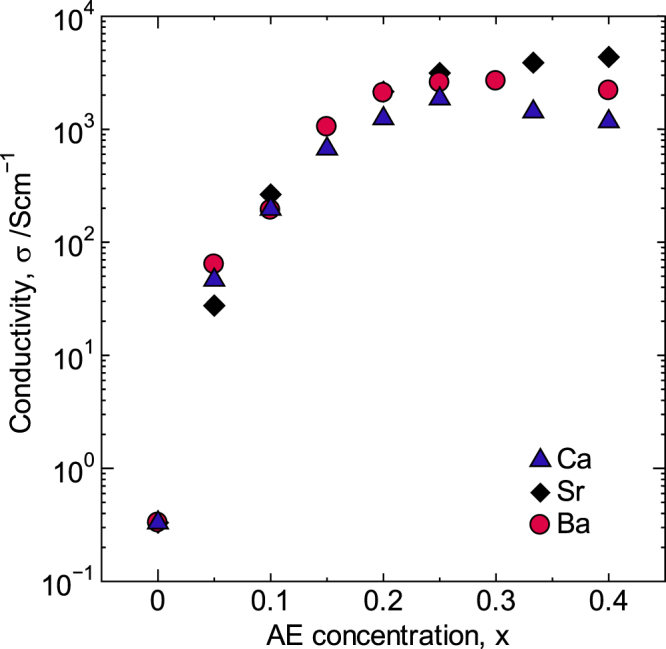
Electrical conductivity of La_1−*x*_AE_*x*_CoO_3_ at R.T. Reproduced from [6] by permission of The Royal Society of Chemistry.

The electrical conductivity (*σ*) for the Sr-doped material was the highest among the oxides doped with the three different AE elements when *x* ≥ 0.25. Notably, the Co–O bond lengths were also the shortest in this composition range. These results suggested a relation between *σ* and the Co–O bond length. Figure 2 shows the AE concentration dependence of the *σ* values obtained at R.T. for 0 ≤ *x* ≤ 0.40. In the 0 ≤ *x* ≤ 0.20 range, the *σ* value increased four orders of magnitude as the AE concentration increased, although there was no significant change in *σ* as a function of the ionic radius of the AE element. On the other hand, the highest values of *σ* for the Sr-doped system were observed when 0.20 < *x* ≤ 0.40, with a maximum of 4.4 × 10^3^ S cm^−1^ reached at *x* = 0.40. This value is the highest level compared with any previously reported value [18, 25]. The conductivities for the Ca- and Ba-doped materials, however, reached maximum values of 1.9 × 10^3^ S cm^−1^ at *x* = 0.25 and 2.7 × 10^3^ S cm^−1^ at *x* = 0.30, respectively. Notably, these materials are potential replacements for metal electrodes and wiring.

The carrier concentration (*n*) and carrier mobility (*μ*) determined via Hall-effect measurements at R.T. have been previously reported for the AE-doped LaCoO_3_ systems [6]. The values of *n* increased over two orders of magnitude because of AE doping, initially rising steeply with the increasing AE concentration in the 0 ≤ *x* ≤ 0.2 range, then becoming saturated in the 0.20 < *x* ≤ 0.40 range and finally reaching maximum values of 7.2 × 10^21^ cm^3^ (*x* = 0.20), 8.4 × 10^21^ cm^3^ (*x* = 0.36) and 6.6 × 10^21^ cm^3^ (*x* = 0.30) for Ca, Sr and Ba, respectively. There was no significant variation in the *n* values as a function of the ionic radius of the AE element, except when *x* = 0.40. Because the *n* values depended on the AE concentration and not on the AE ionic radius, it can be concluded that no correlation existed between the carrier concentration and the crystal structure consisting of the CoO_6_ octahedra.

The *μ* value often decreases because of element doping or element substitution when there is no large modification in the crystal structure. This change in value can be attributed to a decrease in the relaxation time caused by lattice defects, as shown by the equation as follows:1
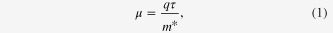
where *μ*, *q*, *τ* and *m*∗ are the carrier mobility, electronic charge, relaxation time and DOS effective mass, respectively. However, the current system did not obey this relation; rather, *μ* increased two orders of magnitude as the AE concentration increased for *x* ≤ 0.20, despite doping. In addition, the average ionic radius at the A-site did not influence the *μ* value in this range. On the other hand, the *μ* value for the Sr-doped system was the highest of the systems doped with the three AE elements for 0.25 ≤ *x* ≤ 0.40. Interestingly, in this concentration range, the values for *σ* and *μ* were the largest, whereas those for the Co–O bond length were the smallest for all three AE-doped materials.

### 3.4. Relation between the mobility and the crystal structure of AE-doped LaCoO_3_

**Figure 3.**
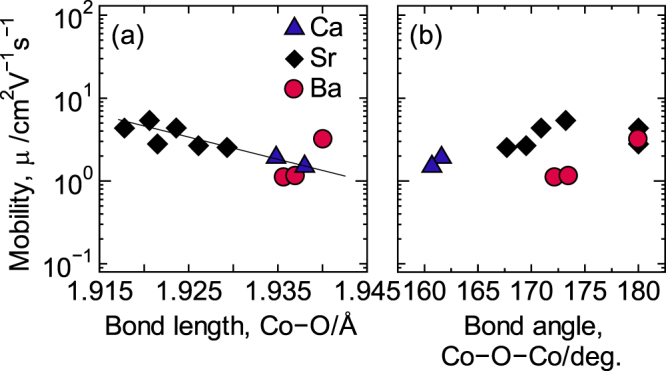
The dependence of the mobility to (a) the Co−O bond angle and (b) the Co−O−Co bond angle. Reproduced from [6] by permission of The Royal Society of Chemistry.

Generally, the *μ* value increases when the Co–O–Co bond angle gets closer to 180° due to the increased overlap and strong interactions between the Co(3*d*)–O(2*p*) orbitals, leading to an increase in the relaxation time [27]. However, while the Co–O–Co bond angle did vary as a function of the average ionic radius at the A-site, the *μ* value did not. On the basis of these results, it can be concluded that no relation existed between *μ* and the Co–O–Co bond angle, as shown in figure 3(b). Consequently, the values for *σ* and *μ* cannot be improved by increasing the Co–O–Co bond angle to 180° in this system. In contrast, a relation between *μ* and the Co–O bond length was observed, as shown in figure 3(a). The *μ* value increased as the Co–O bond length decreased. Therefore, the variation in the behavior of *μ* as a function of the AE concentration when *x* ≥ 0.25 is presumed to be due to the difference in the Co–O bond lengths. This result coincides with a previous report concerning La_1−*x*_Sr_*x*_CoO_3_ [33], although the behavior of the Co–O bond length as a function of the Sr concentration is not the same.

## 4. Electron transport properties of Sr-doped LaCoO_3_ [7]

### 4.1. Applications of La_1−x_Sr_x_CoO_3±*δ*_

La_1−*x*_Sr_*x*_CoO_3±*δ*_ has properties suited to various fields. Its use as a SOFC cathode [22, 23], a thermoelectric material [17, 20, 21, 34], an oxygen-permeable membrane [24, 25] and in nonvolatile memory [19] has been investigated. In addition, its unusual magnetism [35–39] and high oxide-ion conduction [40–42] have received much attention from a basic research perspective. These properties can be attributed directly to the effects of Sr doping at the La-site. Therefore, the investigation of the electronic conduction in La_1−*x*_Sr_*x*_CoO_3±*δ*_ is meaningful in many areas of research. A metal–insulator transition is one of the typical effects that results from Sr doping at the La-sites in LaCoO_3_ [17, 26]. Iwasaki *et al* [17] showed that La_1−*x*_Sr_*x*_CoO_3_ with *x* ≤ 0.30 is metallic in the temperature range of 100–1100 K. The metal–insulator transition originates from the spin-state transition of the cobalt ions in LaCoO_3_ [28, 33]. However, despite numerous studies of various aspects of La_1−*x*_Sr_*x*_CoO_3±*δ*_, the investigation of its high electrical conductivity has rarely been reported, and the mechanism remains unclear. Therefore, a systematic evaluation of the electronic conduction across the complete range of La/Sr ratios was performed.

### 4.2. Crystal structure of La_1−x_Sr_x_CoO_3±*δ*_ as a function of the La/Sr ratio

La_1−*x*_Sr_*x*_CoO_3±*δ*_ was identified as a perovskite-type structure based on a rhombohedral lattice for 0 ≤ *x* ≤ 0.80, as previously reported in detail [7]. In this concentration range, the main XRD peaks near 33° were initially combined and then sharpened with a shift to lower angles as the Sr concentration increased. When *x* ≥ 0.90, a brownmillerite-type structure was identified as a second phase, and a single phase of this structure occurred at *x* = 1.0, indicating that La_1−*x*_Sr_*x*_CoO_3±*δ*_ did not form a complete solid solution over the entire composition range.

**Figure 4.**
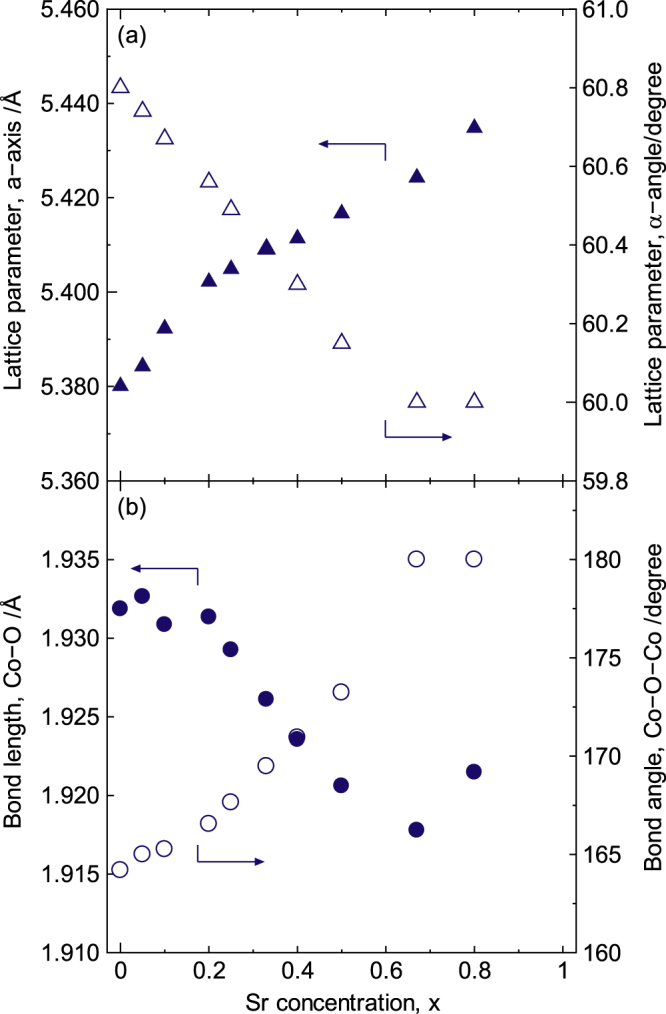
Lattice parameters of La_1−*x*_Sr_*x*_CoO_3±*δ*_. (a) Lattice angle and lattice length of the rhombohedral unit cell and the (b) Co–O–Co bond angle and Co–O bond length of the CoO_6_ octahedron. Reproduced from [7] by permission of The Royal Society of Chemistry.

The lattice parameters for La_1−*x*_Sr_*x*_CoO_3±*δ*_ optimized via Rietveld refinement [31] are shown in figure 4. The space group *R-*3*c* was used for the convergence in this refinement, although both *R-*3*c* and *I*2/*a* have been proposed for this system [28–30, 43]. The lattice angle (*α*) and the Co–O–Co bond angle approached 60° and 180°, respectively, with increasing Sr concentration for 0 ≤ *x* < 2/3; both then became saturated at *x* ≥ 2/3, indicating that the unit cell for La_1−*x*_Sr_*x*_CoO_3±*δ*_ changed from rhombohedral to cubic at *x* =2/3. This result suggests that the electrostatic attractive force between Co and O intensified as the Co–O–Co bond angle approached 180°. In this range, lattice length (*a*) also increased with the increasing Sr concentration, although the Co–O bond length decreased for *x* < 2/3.

### 4.3. Electrical conductivity and Seebeck coefficient of La_1−x_Sr_x_CoO_3±*δ*_

**Figure 5.**
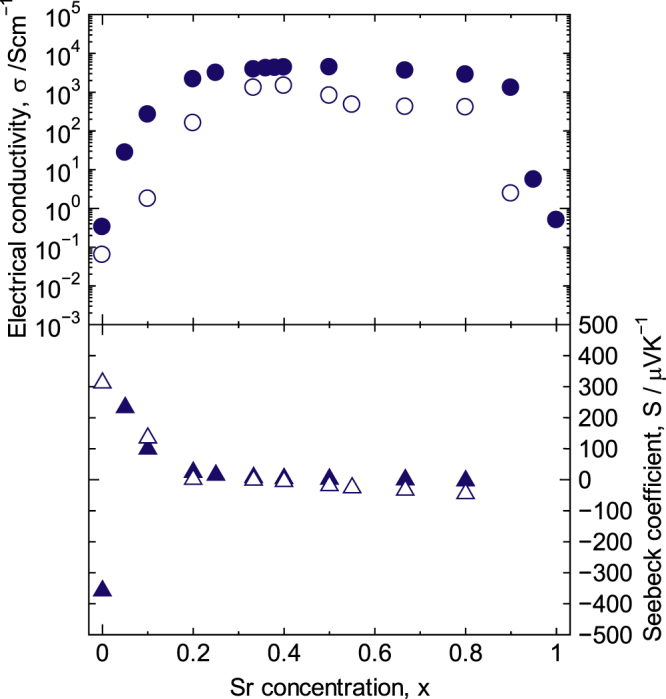
Electrical conductivity and Seebeck coefficient for 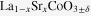 (solid symbol) and La_1−*x*_Sr_*x*_MnO_3±*δ*_ (open symbol) at R.T. [7]. The data of La_1−*x*_Sr_*x*_CoO_3±*δ*_ are reproduced by permission of The Royal Society of Chemistry.

The electrical conductivity (*σ*) and the Seebeck coefficient (*S*) of La_1−x_Sr_x_CoO_3±*δ*_ at R.T. as a function of the Sr concentration are shown in figure 5; neither has a relation with the lattice parameters. The *σ* value increased four orders of magnitude as the Sr concentration increased in the range of 0 ≤ *x* < 0.20 and then continued to increase gradually in the range of 0.20 ≤ *x* ≤ 0.50. The maximum conductivity of 4.4 × 10^3^ S cm^−1^ was observed at *x* = 0.50. In the *x* > 0.50 concentration range, the *σ* value remained high until *x* = 0.80 and then decreased steeply for *x* > 0.80. This decrease in conductivity in the *x* > 0.80 range is attributed to the partial formation of a brownmillerite impurity phase. Overall, for the 0.20 ≤ *x* ≤ 0.80 range, the *σ* value was nearly independent of the Sr concentration. Such insensitivity to the composition ratio is an advantage for industrial applications.

The maximum value of *S* for La_1−*x*_Sr_*x*_CoO_3±*δ*_ in the concentration range of 0.05 ≤ *x* ≤ 0.80 was 233 *μ*VK^−1^ at *x* = 0.05, whereas the minimum *S* value of −236 *μ*VK^−1^ was observed at *x* = 0. In this range, *S* gradually decreased as the Sr concentration increased and approached zero when 0.5 ≤ *x* ≤ 0.80. The sign of *S* reversed from negative to positive in the initial stage of Sr substitution at concentrations between *x* = 0 and 0.05, i.e. the La_1−*x*_Sr_*x*_CoO_3±*δ*_ (0.05 ≤ *x* ≤ 0.80) investigated in this study was a p-type system, whereas pure LaCoO_3_ is an n-type material, although the sign of LaCoO_3_ remains to be further discussed [17, 20, 44]. On the basis of these results, the carriers resulting from the nonstoichiometry are believed to become dominant in weakly doped oxides such as pure LaCoO_3_.

### 4.4. Oxygen content and valence of Co in La_1−x_Sr_x_CoO_3±*δ*_

**Figure 6.**
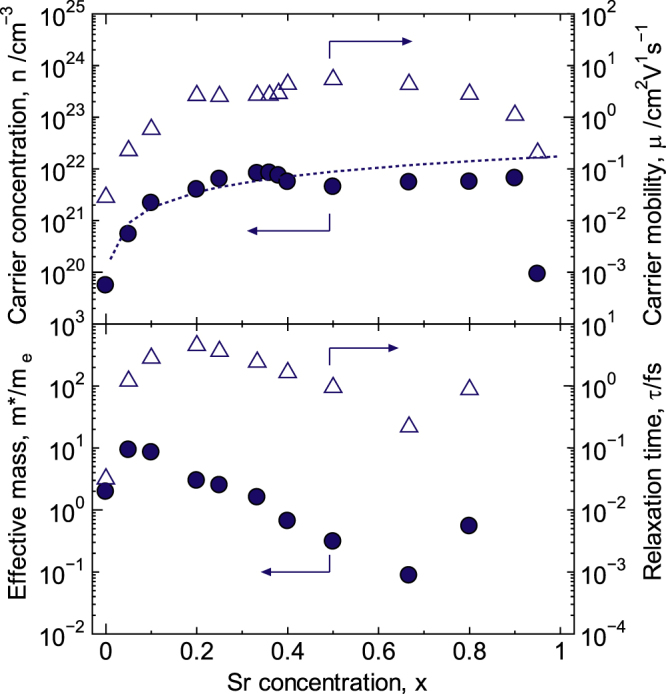
Carrier concentration, mobility, effective mass and relaxation time for La_1−*x*_Sr_*x*_CoO_3±*δ*_ at R.T. The dotted line indicates the theoretical value of the carrier concentration. Reproduced from [7] by permission of The Royal Society of Chemistry.

The p–n transition between *x* = 0 and 0.05 was also observed during Hall-effect measurements. The Hall coefficient reversed from negative to positive between *x* = 0 and 0.05. This result agrees with the behavior of *S*. This transition is caused by the increase in hole concentration by Sr doping to the La-site. Figure 6 shows the carrier concentration (*n*) and the carrier mobility (*μ*) for La_1−*x*_Sr_*x*_CoO_3±*δ*_ at R.T. The *n* value increased two orders of magnitude as the Sr concentration increased in the range of 0 ≤ *x* ≤ 0.36 and reached a maximum of 8.4 × 10^21^ cm^−3^. According to equation (2) (Kröger–Vink notation), the *n* value should continue to increase with the increasing Sr concentration if Sr is completely substituted into the La-sites with additional oxygen incorporation. This theoretical curve for *n* is shown as the dotted line in figure 6. The theoretical value was calculated using the Sr concentration and lattice volume because the hole concentration [*h*^·^] is equal to the Sr concentration [Sr′_La_] when the above condition is satisfied2
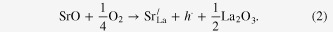



It is likely that conventional hole doping occurs for 0 < *x* ≤ 0.20 because the observed value coincides with the theoretical value. However, the observed and theoretical values do not coincide for *x* ≥ 0.25. Rather, the observed value exceeds the theoretical value in the concentration range of 0.25 ≤ *x* ≤ 0.38, whereas the theoretical value exceeds the observed value in the concentration range of 0.40 ≤ *x* ≤ 0.80. This variation in behavior is believed to originate from the nonstoichiometric composition. The defect equilibrium with respect to cation and oxygen vacancies can be expressed using equations (3) and (4), respectively, as follows:3
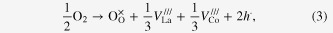

4
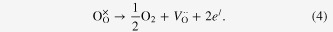



**Figure 7.**
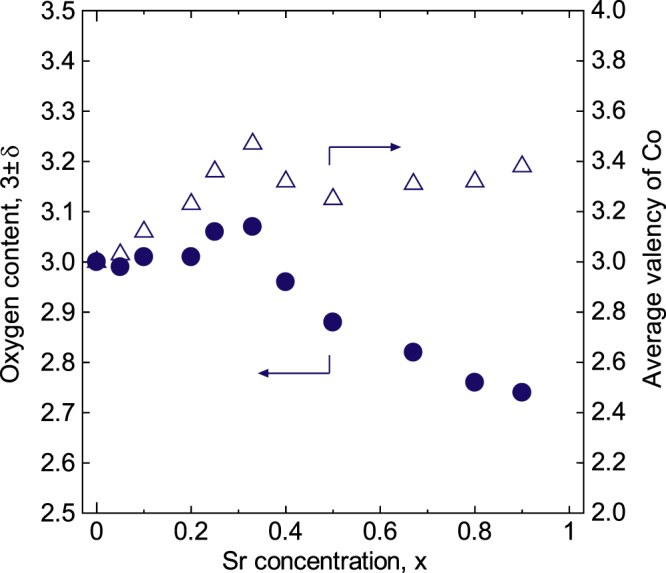
Oxygen content and average valence of Co for 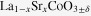 at R.T. Reproduced from [7] by permission of The Royal Society of Chemistry.

Holes are generated by the cation vacancies, whereas electrons are generated by the oxygen vacancies. Likely, *n* values greater than the theoretical value are realized because of the high total number of holes generated due to both Sr doping and the cation deficiency in the 0.25 ≤ *x* ≤ 0.38 concentration range, whereas *n* values less than the theoretical value occur as a result of the annihilation of the holes generated due to Sr doping and the electrons generated by the oxygen deficiency in the 0.40 ≤ *x* ≤ 0.80 concentration range. The reported values for oxygen nonstoichiometry are not always consistent because of the differences in the synthetic conditions and measurement atmospheres [17, 20, 45, 46]. Therefore, the oxygen nonstoichiometry was estimated to be 3 ± *δ* by assuming that the difference between the theoretical and experimental values for the carrier concentration corresponds to the number of electrons generated by the oxygen deficiency, as shown in figure 7. For this estimate, cation-deficient compositions were regarded as being oxygen-excessive. La_1−*x*_Sr_*x*_CoO_3±*δ*_ had an oxygen-excess composition for 0.20 < *x* < 0.40 and was oxygen deficient for *x* ≥ 0.40. In addition, the oxygen content evaluated via iodometric titration was 2.99, 2.98 and 2.78 for *x* = 0.20, 0.40 and 0.80, respectively. These values are consistent with those estimated from the carrier concentration. In addition, the carrier concentration was 5.6 × 10^19^ cm^−3^, and the Hall coefficient was negative at *x* = 0, i.e. non-doped LaCoO_3_. This means that the electrons are generated by the oxygen deficiency at *x* = 0. The carriers resulting from the nonstoichiometry are believed to become dominant in weakly doped oxides such as pure LaCoO_3_.

Moreover, the average valence of Co was more than three over the entire concentration range (0 ≤ *x* ≤ 1.0), suggesting the presence of Co^4+^, which is an anomalous valence. The average valence of Co means the itinerant carriers around Co ions. So, the behavior of the average valence of Co and the carrier concentration are related to each other. Both reached the maximum at *x* = 0.33–0.36. However, the maximum electrical conductivity was not observed at *x* = 0.33–0.36 because the maximum carrier mobility was observed at *x* = 0.50. To obtain the high electrical conductivity, both the carrier concentration and the carrier mobility should be enhanced. As a result, the carrier concentration, the oxygen nonstoichiometry and the average valence of Co were not connected with the lattice parameters.

### 4.5. Analysis of the carrier mobility in La_1−x_Sr_x_CoO_3±*δ*_

The behavior of the carrier mobility (*μ*) in La_1−x_Sr_x_CoO_3±*δ*_ as a function of the Sr concentration is also interesting, as shown in figure 6. The *μ* value generally decreases on element doping or element substitution because the relaxation time decreases owing to carrier scattering resulting from lattice defects. However, the *μ* value for this system increased two orders of magnitude as the Sr concentration increased for *x* < 0.50, reaching a maximum of 5.8 cm^2^ V^−1^ s^−1^ at *x* = 0.50. Therefore, to further understand the behavior of the 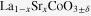 system, the DOS effective mass (*m*∗) and the relaxation time (*τ*) were calculated using the following equations [47]:5
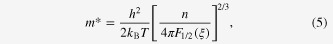

6
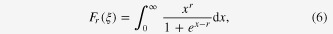

7
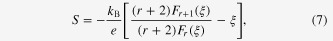

8
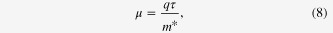
where *h*, *k*_B_, *T*, *F*_*r*_, *ξ* and *r* are Planck’s constant, Boltzmann’s constant, the absolute temperature, the Fermi integral, the chemical potential and the carrier scattering parameter of the relaxation time, respectively. In this derivation, acoustic-phonon scattering (*r* = 0) was assumed in the 0.05 ≤ *x* ≤ 0.80 range, and ionized impurity scattering (*r* = 2) was assumed at *x* = 0. The *m*∗ and *τ* values are shown in figure 6. Considering the absolute values for *m*∗ and *τ*, the increase in *μ* for 0 ≤ *x* < 0.2 was dominated by *τ*, whereas the maximum value for *μ* at *x* = 0.50 was dominated by *m*∗ (= 0.31 m_e_). Moreover, the behavior of *m*∗ as a function of the Sr concentration was analogous to that of the Co–O bond length, suggesting that the decrease in *m*∗ occurred as a result of the decrease in the Co–O bond length.

### 4.6. Relation between the DOS and the effective mass

**Figure 8.**
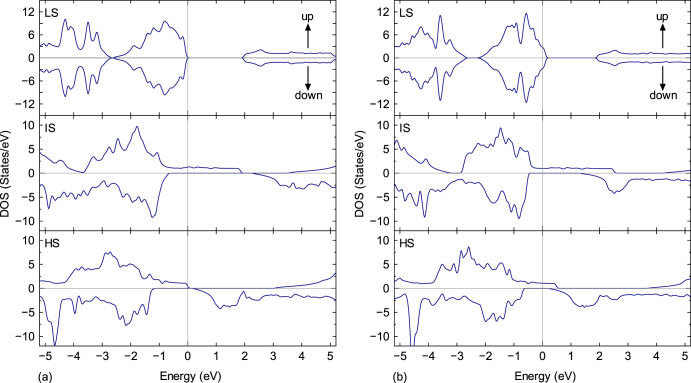
DOS calculated by the GGA + U method. (a) LaCoO_3_ and (b) La_0.5_Sr_0.5_CoO_3_. Reproduced from [7] by permission of The Royal Society of Chemistry.

The DOS near the Fermi level involves important information for *m*∗ of carriers because it is related to the band curvature and the number of available states for carriers. To investigate the decrease in *m*∗ with the increasing Sr concentration, the DOS was calculated using first-principles calculations, as shown in figure 8 [48–50]. Spin-state transitions of the cobalt ions in LaCoO_3_ occur with an increase in temperature or with Sr substitution [51, 52]. These transitions have been interpreted based on a simple model assuming low-spin (LS, *S* = 0), intermediate-spin (IS, *S* = 1) and high-spin (HS, *S* = 2) states of the cobalt ions. In the ground state, the cobalt ions in LaCoO_3_ are in the nonmagnetic LS state. As the temperature increases, two spin-state transitions occur with transition temperatures of approximately 100 K and 500 K [32, 35, 53, 54]. Similarly, the introduction of holes due to Sr doping into the La-sites also induces spin-state transitions and magnetism [52, 55]. To clarify the influence of the spin-state transition on the DOS, the DOS for the three spin states were calculated for the fixed material compositions of LaCoO_3_ and La_0.5_Sr_0.5_CoO_3_. For the three states in each composition, the valence band gradually broadened and shifted to the lower energy side as the spin state increased. For the two compositions at each state, the value of the Fermi energy (*E*_F_) shifted to the low energy side due to hole doping, although no difference in the DOS was observed. In addition, the LS state in each composition had a clear band gap of approximately 2 eV. Moreover, the *E*_F_ of La_0.5_Sr_0.5_CoO_3_ was in the valence band as a result of hole doping, whereas the *E*_F_ of LaCoO_3_ was in the band gap near the valence-band maximum. In contrast, the IS and HS states were extended beyond the *E*_F_ because of the formation of ‘tails’ in the up-spin direction. Thus, the electronic structures near the *E*_F_ were found to be substantially different for the LS, IS and HS states, suggesting a significant difference in the values for *m*∗ and *μ*.

To reveal the effects of the spin states on *m*∗, the relation between the carrier concentration (*n*) and the effective mass (*m*∗) was then calculated from the DOS for each of the three states of LaCoO_3_ and La_0.5_Sr_0.5_CoO_3_ using the BoltzTraP code, which is based on the semiclassical Boltzmann theory [56], as shown previously [7]. The values for *m*∗ for the LS states were larger than those for the IS and HS states over the entire range. In addition, the *m*∗ values for the LS state of LaCoO_3_ were larger than those for the LS state of La_0.5_Sr_0.5_CoO_3_. A decrease in the *m*∗ value was observed following the spin-state transitions from the LS state to the IS and HS states. Unfortunately, this analysis did not clarify the spin states for pure LaCoO_3_ (100 < *T* < 500 K) or Sr-doped LaCoO_3_, and they remain a matter of debate [32, 35, 52–55]. Notably, although the spin states for these two complexes are either IS or mixed IS/HS states, the values for *m*∗ for both systems decreased steeply to those of the LS states.

### 4.7. Section summary

The high *σ* value of 4.4 × 10^3^ S cm^−1^ for La_0.5_Sr_0.5_CoO_3_ is attributed to its high *n* value of 4.5 × 10^21^ cm^−3^ and small *m*∗ value of 0.31 m_e_. The observed decrease in the *m*∗ value originates from the spin-state transition that occurs because of Sr doping. A certain correlation between this transition and a decrease in the Co–O bond length with the increasing Sr concentration was also suggested. Although La_1−*x*_Sr_*x*_CoO_3±*δ*_ is an attractive material with respect to its electronic conduction, the elimination of oxygen with the increasing temperature is an issue for its application in ceramic-based products. The weight of La_0.5_Sr_0.5_CoO_3−*δ*_ decreased by approximately 2.8 wt% as the temperature increased from R.T. to 1173 K, and this weight loss was shown to correspond to oxygen elimination [7, 42], which is unsuitable for oxide-based electrodes and wiring.

## 5. n-type conduction in La_1−*x*_Sr_*x*_MnO_3±*δ*_ [9]

### 5.1. Need for n-type oxide electrodes

Both n-type and p-type oxides are required to effectively utilize the electronic conduction of oxides. If electrodes, wiring, p–n junctions and ohmic contacts can all be formed using oxides at high temperature, innovations in the ceramics industry will be realized. However, n-type oxides are generally unstable at high temperatures in air or other oxidizing atmospheres because an oxygen deficiency is required to generate electrons, whereas p-type oxides, which should have an excess of oxygen to generate holes, are stable in air. Consequently, an important innovation in the ceramics field would be the development of n-type oxide ceramic products with excellent performance that are stable at high temperatures in air. Therefore, we focused on the perovskite-type oxide La_1−*x*_Sr_*x*_MnO_3±*δ*_, which is an n-type oxide in the Sr concentration range of 0.02 ≤ *x* ≤ 0.50 above 400 K [57, 58]. This oxide should be stable in nonstoichiometric compositions and should also be insensitive to changes in the partial pressure of oxygen due to the three stable valences of Mn (i.e. Mn^2+^, Mn^3+^ and Mn^4+^).

La_1−*x*_Sr_*x*_MnO_3±*δ*_ exhibits some interesting properties that are relevant for specialized applications. While 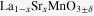 is understood to be a p-type oxide in many cases, the boundary between the n-type and p-type is not always consistent in previous reports [57–59]. It is a promising SOFC cathode material because it exhibits mixed conduction, is resistant to reduction and has a moderate coefficient of thermal expansion [60, 61]. La_0.8_Sr_0.2_MnO_3_ synthesized using an aqueous solution technique is often used for SOFCs. Mizusaki *et al* showed that La_1−*x*_Sr_*x*_MnO_3_ (0 ≤ *x* ≤ 0.20), which is synthesized from an aqueous acetate solution, is p-type at the P_O2_ = 1 bar in the temperature range of 273 ≤ *T* ≤ 1373 K. Tokura *et al* found that La_1−*x*_Sr_*x*_MnO_3_ single crystals grown via the floating-zone (FZ) method displayed a colossal magnetoresistance (CMR) effect [62, 63]. They also reported the electronic phase diagram with respect to the magnetic transition for compositions with *x* ≤ 0.60 [62]. The ferroelectricity of La_1−*x*_Sr_*x*_MnO_3_ occurs because of double exchange interactions [62–66], and the contribution of ligand holes has been suggested [67, 68]. The magnetism of La_1−*x*_Sr_*x*_MnO_3_ has received much attention since the discovery of the CMR effect [69–71]. However, in most cases, these studies were performed at low temperatures for *x* ≤ 0.50; thus, La_1−*x*_Sr_*x*_MnO_3_ has been considered a p-type system. In contrast, in the present study, the focus was on the n-type region of La_1−*x*_Sr_*x*_MnO_3±*δ*_ (0 ≤ *x* ≤ 1.0), and the electrical properties of this material were systematically investigated from R.T. to 1173 K in air.

### 5.2. Relation between the electrical conductivity and crystal system for La_1−x_Sr_x_MnO_3±*δ*_

In the 0 ≤ *x* ≤ 0.80 Sr concentration range, the XRD patterns of La_1−*x*_Sr_*x*_MnO_3±*δ*_ were all assigned as perovskite phases, and other peaks were not detected. In contrast, the patterns for the Sr concentration range of 0.90 ≤ *x* ≤ 1.0 were different from those of a simple perovskite-type oxide. These patterns were indexed based on a hexagonal cell in the *P*63/*mmc* space group, which involves face-sharing MnO_6_ octahedra [72–74]. La_0.1_Sr_0.9_MnO_3±*δ*_ and SrMnO_3_ correspond to six- and four-layered BaMnO_3_ structures, respectively [75]. The space group changed as follows: *Pnma* (0 ≤ *x* ≤ 0.1) → *R-*3*c* (0.20 ≤ *x* ≤ 0.40) → *I*4/*mcm* (0.50 ≤ *x* ≤ 0.67) → *Pm*3 *m* (*x* = 0.80).

Figure 5 shows the Sr content dependence of the electrical conductivity (*σ*) and the Seebeck coefficient (*S*). The *σ* value for La_1−*x*_Sr_*x*_MnO_3±*δ*_ was smaller than that for 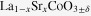 over the entire La/Sr ratio range. In addition, the *σ* value for La_1−*x*_Sr_*x*_MnO_3±*δ*_ reached a maximum of 1.5 × 10^3^ S cm^−1^ at *x* = 0.40, for which the space group was *R-*3*c*. Notably, the maximum value of *σ* was observed at the same Sr concentration that resulted in the shortest length for the Mn–O bond in the *R-*3*c* region (0.20 ≤ *x* ≤ 0.40). Interestingly, in this region, the Mn–O bond length decreased as the Sr concentration increased. However, the behavior of the *σ* value cannot be understood only by the change in the Mn–O bond length because the crystal system for La_1−*x*_Sr_*x*_MnO_3±*δ*_ changes drastically with the Sr concentration over the entire concentration range.

### 5.3. Hall-effect measurements for La_1−x_Sr_x_MnO_3±*δ*_ at R.T.

The *S* value changed from positive to negative at approximately *x* = 0.20. This behavior corresponds to the Hall coefficient (*R*_H_). *R*_H_ was negative of −0.02 < *R*_H_ < 0 cm^3^ C^−1^ in the concentration range of 0.20 ≤ *x* ≤ 0.80, while it was 0.75 cm^3^ C^−1^ at *x* = 0.10. Notably, the *x* value at the p–n transition (0.20) did not agree with the previously reported values [57–59] for La_1−*x*_Sr_*x*_MnO_3±*δ*_. The differences in the synthetic methods and reaction conditions for the various samples are believed to be the cause, as was the case for 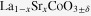.

Importantly, a linear relation between the Hall resistance and the applied magnetic field (*B*) for *x* = 0.40 in the range of 4.0 × 10^−2^ ≤ *B* ≤ 5.0 × 10^−1^ T was observed. Thus, the values of *n*, *μ*, *m*∗ and *τ* for La_1−x_Sr_x_MnO_3±*δ*_ with *x* = 0.40, evaluated via Hall-effect measurements at *B* = 3.0 × 10^−1^ (T) and calculated using equations (5)–(8), were 1.5 × 10^21^ cm^−3^, 5.0 cm^2^ V^−1 ^s^−1^, 0.11 m_e_ and 0.30 fs, respectively. In this derivation, ionized impurity scattering (*r* = 2) was assumed because *μ* increased as a function of *T*^1.2^. Accordingly, the high *σ* value of 1.5 × 10^3^ S cm^−1^ at *x* = 0.40 is because of a high carrier concentration of 1.5 × 10^21^ cm^−3^ and a small effective mass of 0.11 m_e_, as was the case for 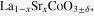 and this composition is consistent with the smallest Mn–O bond length in the *R-*3*c* region.

### 5.4. Temperature dependence of σ as a function of the La/Sr ratio for La_1−x_Sr_x_MnO_3±*δ*_

**Figure 9.**
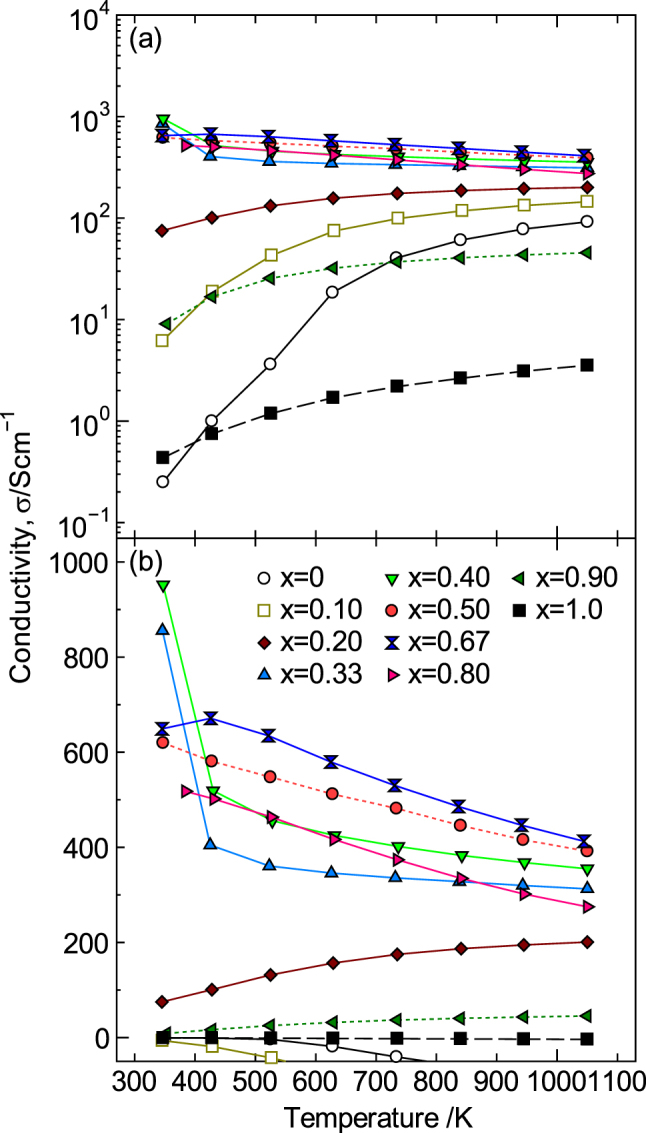
(a) Temperature dependence of the electrical conductivity, *σ*, for La_1−*x*_Sr_*x*_MnO_3±*δ*_ (0 ≤ *x* ≤ 1.0) and (b) enlarged view with a linear scale for *σ* ≤ 1000 S cm^−1^. Reproduced from [9] by permission of The Royal Society of Chemistry.

Figure 9 shows the *σ* values for La_1−*x*_Sr_*x*_MnO_3±*δ*_ measured between R.T. and 1073 K for various Sr concentrations. The largest value of *σ* was observed between 423 and 1073 K when *x* = 0.67, but samples with 0.33 ≤ *x* ≤ 0.80 also exhibited high *σ* values. On the other hand, the largest value of *σ* at 300–423 K was obtained when *x* = 0.40. In addition, the *σ* values for *x* = 0.33, 0.40 and 0.50 exhibited metallic behavior over the entire temperature range, i.e. *σ* decreased as the temperature increased, except for an anomaly that occurred between 373–473 K. This anomaly is due to a ferromagnetic to paramagnetic transition [62]. In contrast, the *σ* values for *x* = 0.55, 0.67 and 0.80 exhibited a metal–insulator transition at approximately 373 K (semiconducting behavior appeared at *T* < 373 K, whereas metallic behavior was observed at *T* ≥ 373 K). In addition, the temperature of the metal–insulator transition increased as the Sr content increased. Consequently, both the largest *σ* value between 423 K and 1073 K and the lowest rate of the change of the *σ* value with the temperature were observed when *x* = 0.67, which is therefore a suitable composition for use as oxide electrodes and wiring that are operated at high temperatures in air.

**Figure 10.**
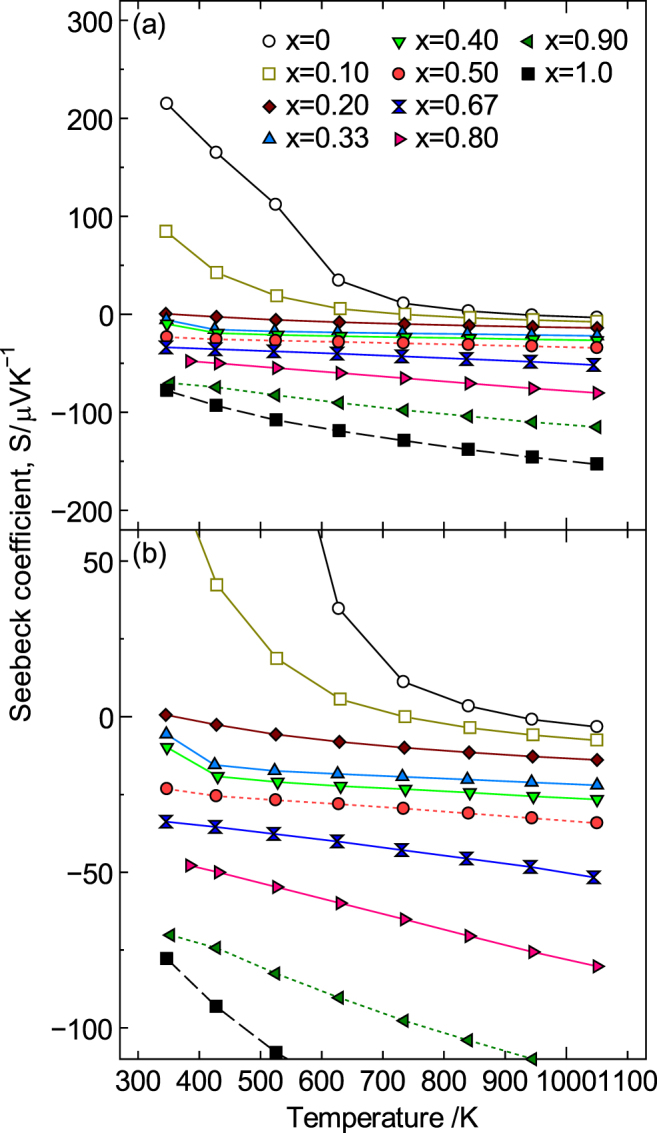
(a) Temperature dependence of the Seebeck coefficient, *S*, for La_1−*x*_Sr_*x*_MnO_3±*δ*_ (0 ≤ *x* ≤ 1.0) and (b) enlarged view between −100 ≤ *S* ≤ 50 *μ*VK^−1^. Reproduced from [9] by permission of The Royal Society of Chemistry.

Figure 10 shows the temperature dependence of *S*. In contrast to the values for *σ*, the Seebeck coefficient systematically decreased as the temperature and Sr content increased, and the sign of *S* reversed from positive to negative, although the pure LaMnO_3_ was p-type (*S* > 0). For *x* ≥ 0.33, the *S* value was negative over the entire temperature range, indicating that the major conduction carriers were electrons. In particular, the decrease in *S* became distinct for 0.50 ≤ *x* ≤ 0.80 and then more remarkable for 0.80 < *x* ≤ 1.0 and reached a minimum value of −152 *μ*V K^−1^ at *x* = 1.0 and 1073 K. However, changes in the carrier concentration cannot explain the decrease in *S* for 0.50 ≤ *x* ≤ 1.0, i.e. the increase in the absolute value of *S* (|*S*|) because |*S*| increased as the temperature increased. Generally, |*S*| for a semiconductor is believed to decrease as the temperature increases because the carriers increase due to thermal excitation. In addition, the carrier concentration of a metal is insensitive to temperature because carriers are temperature-independent. Therefore, |*S*| should not increase with temperature based on the Mott equation, as follows [77, 78]:9
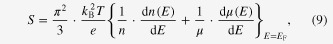
where *k*_B_, *e*, *n* and *μ* are the Boltzmann constant, electron charge, carrier concentration and carrier mobility, respectively. In addition, |*S*| depends on the energy derivative of the DOS at the Fermi level [78]. Thus, the behavior of *S* in 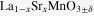 is not due to *n* but to *μ*, i.e. the DOS effective mass (*m*∗). The semiconductors with *x* = 0.90 and 1.0 are particularly interesting because |*S*| increased as the temperature increased. As mentioned above, *x* = 0.90 and 1.0 are BaMnO_3_-type structures with Mn^4+^ at the B-site. Hishida *et al* [76] performed an x-ray photoemission spectroscopy (XPS) study of the La_1−*x*_Sr_*x*_MnO_3±*δ*_ synthesized in this study and found that the intensity ratio of the Mn^4+^ peak in the Mn 2*p*3/2 spectrum began to increase as the Sr content increased from 0.50 to 0.80 and then increased more steeply when the Sr concentration increased from 0.80 to 1.0. This behavior of the Mn^4+^ concentration versus the Sr content coincides with that of the *S* value. Accordingly, the behavior of |*S*| for 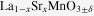 most likely originates from the increase in the *m*∗ of the electrons, which results from the greater quantity of Mn^4+^ induced by the increase in the Sr content. The valence change of Mn (3+ → 4+) contributes to both *n* and *m*∗. Therefore, the increase in *m*∗ and the decrease in *n* can be concluded to occur as a result of the increase in Mn^4+^, and |*S*| increased as a result. Note that when *x* = 0.67, which is a suitable Sr concentration for oxide electrodes and wiring, La_1−*x*_Sr_*x*_MnO_3*±δ*_ is clearly n-type over the entire temperature range.

In contrast, the behavior of the Mn^4+^ concentration versus the Sr content does not coincide with that of the *σ* value. The concentration for itinerant carriers around Mn related to the *σ* value cannot be simply explained by the valence change of Mn because both Mn^3+^ and Mn ^4+^ are stable in this system.

### 5.5. Durability of La_1−x_Sr_x_MnO_3±*δ*_ at high temperatures in air

Generally, n-type oxides, which have electrons, are believed to be thermodynamically unstable at high temperatures in air because they are oxygen deficient, according to equation (4), i.e. as oxygen deficiencies are annihilated by oxidation at high temperatures in air, fewer itinerant electrons are available. In other words, n-type oxides become unstable because of an increase in the cation vacancies at high temperatures in air.

**Figure 11.**
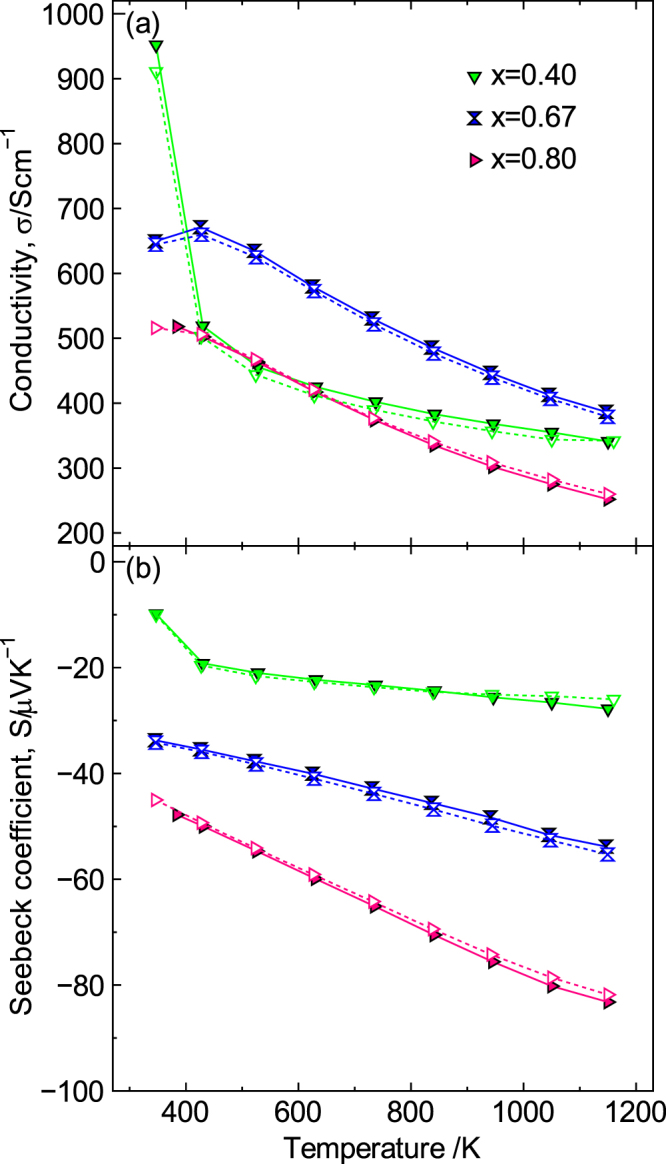
Stability of La_1−*x*_Sr_*x*_MnO_3±*δ*_ (*x* = 0.40, 0.67, 0.80). (a) Conductivity and (b) Seebeck coefficient. The solid line and symbols are before annealing in air at 1273 K for 100 h; the dotted line and open symbols are after annealing at 1273 K for 100 h. Reproduced from [9] by permission of The Royal Society of Chemistry.

Figure 11 shows the durability of La_1−*x*_Sr_*x*_MnO_3±*δ*_ (*x* = 0.40, 0.67 and 0.80) evaluated by comparing the temperature dependence of both *σ* and *S* before and after annealing at 1273 K for 100 h in air. For each composition, annealing had a negligible effect on both *σ* and *S*. Moreover, the *σ* value for *x* = 0.67 barely changed with annealing at 1273 K for 500 h, as reported previously [9]. These data indicate that La_1−*x*_Sr_*x*_MnO_3±*δ*_ has a high phase stability at high temperatures in air, although it is an n-type oxide.

Clearly, the La_1−*x*_Sr_*x*_MnO_3±*δ*_ synthesized in the present study was stable at high temperatures in air. The Mn^3+^ ions associated with the formation of itinerant electrons are believed not to change at high temperatures in air. In other words, the oxygen deficiency and itinerant electrons in 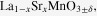 which involve Mn^3+^ and Mn^4+^ states, respectively, do not annihilate due to oxidation. Thus, 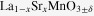 resists oxidation because both Mn^3+^ and Mn^4+^ are thermodynamically stable, as previously described [79].

### 5.6. Section summary

For La_1−*x*_Sr_*x*_MnO_3±*δ*_, the composition with *x* = 0.67, which has the largest *σ* value between 423 and 1073 K and exhibits the lowest rate of change in *σ* with the temperature, is the most suitable for the fabrication of oxide electrodes and wiring that are operated at high temperatures in air. Although it is clearly n-type, this composition of La_1−*x*_Sr_*x*_MnO_3±*δ*_ has a high phase stability up to 1273 K in air. However, the electrical conductivity of La_1−*x*_Sr_*x*_MnO_3±*δ*_ is slightly low (≤800 S cm^−1^) when *T* > 400 K. On the other hand, the high *σ* value of 1.5 × 10^3^ S cm^−1^ at *x* = 0.40 is because of a high carrier concentration of 1.5 × 10^21^ cm^−3^ and a small effective mass of 0.11 m_e_, as is the case for La_1−*x*_Sr_*x*_CoO_3±*δ*_ at R.T., and this composition is consistent with the smallest Mn–O bond length in the *R-*3*c* region.

## 6. Ni doping to the conduction path in LaCoO_3_ [8]

### 6.1. Background on LaCo_1−x_Ni_x_O_3±*δ*_

The chemical doping of La-based perovskite-type oxides is generally performed at the La-sites to inhibit any decrease in the carrier mobility; thus, it does not directly affect the electronic conduction. This type of doping is referred to as modulation doping.

Although there are fewer reports of Ni doping to the Co-sites, which is a conduction path, compared with those of AE doping to La-sites in LaCoO_3_, the control of the electrical conductivity and magnetic properties via Ni doping has been investigated. Li and Li reported that 20 mol% Ni doping led to an increase in the electrical conductivity of LaCo_1−*x*_Ni_*x*_O_3_ to 500 S cm^−1^ at 600 K due to an increase in the carrier concentration and found that the power factor, *S*^2^*σ*, for LaCo_0.9_Ni_0.1_O_3_ was approximately 3.5 times higher than that for pure LaCoO_3_ [80]. Interestingly, although LaCo_1−*x*_Ni_*x*_O_3_ has a high electrical conductivity despite element doping to a conduction path, employing this LaCo_1−*x*_Ni_*x*_O_3_ in oxide electrodes and wiring is difficult because its temperature coefficient of conductivity is very large. Separately, Asai *et al* concluded that Co doping enhances the temperature-independent susceptibility of LaNiO_3_. In addition, glassy ferromagnetism and a very large negative magnetoresistance and metal–insulator transition at *x* = 0.40 have been reported for LaCo_1−*x*_Ni_*x*_O_3_ [81, 82].

Despite its interesting characteristics, the literature contains few reports on LaCo_1−*x*_Ni_*x*_O_3±*δ*_, and the information about physical properties at high temperatures in air is especially insufficient. Therefore, the effects on the electronic conduction of LaCoO_3_ due to Ni doping at Co-sites were verified through the analysis of the crystal and electronic structures of the system at various Ni concentrations.

### 6.2. Crystal structure analysis of LaCo_1−x_Ni_x_O_3±*δ*_

**Figure 12.**
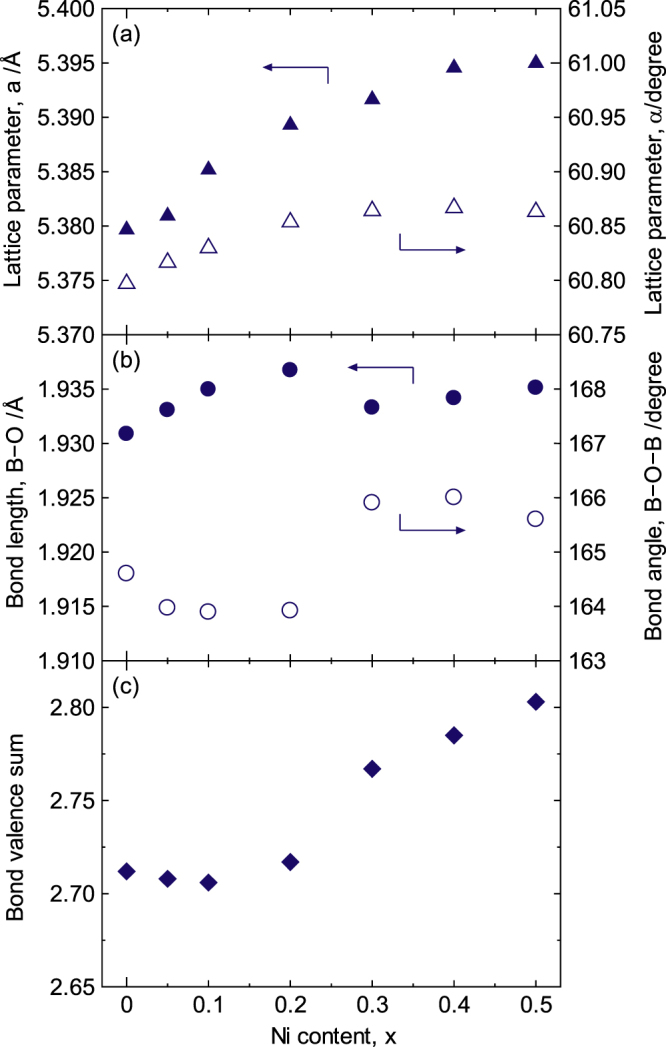
Lattice parameters for LaCo_1−*x*_Ni_*x*_O_3±*δ*_ based on a rhombohedral unit cell. (a) Lattice constants of the *a*-axis and *α* angle, (b) B−O−B bond angle and B−O bond length and (c) bond-valence-sum for B-site ions. Reprinted with permission from [8]. Copyright © 2012 American Chemical Society.

The crystal parameters were refined via Rietveld analysis [31] using the XRD patterns for LaCo_1−*x*_Ni_*x*_O_3±*δ*_ (0 ≤ *x* ≤ 0.5)-sintered bodies and are shown in figure 12. The space group *R-*3*c* based on a rhombohedral lattice [83, 30] was used in this refinement. Both the *a*-axis and *α* angle varied monotonically with the Ni content, whereas the B−O bond length and the B−O−B bond angle were discontinuous and exhibited anomalies between 0.20 < *x* < 0.30. The B−O bond length increased with increasing *x* when 0 ≤ *x* ≤ 0.20, then abruptly decreased when 0.20 < *x* < 0.30 and finally increased again when 0.30 ≤ *x* ≤ 0.50. On the other hand, the B−O−B bond angle sharply increased from 164° to 166° as the Ni concentration increased from 0.20 to 0.30. However, the B−O−B bond angle was expected to decrease with the increasing Ni concentration because the tolerance factor for LaCo_1−*x*_Ni_*x*_O_3±*δ*_ decreased with the increasing *x*. Thus, the increase in the B−O−B bond angle for 0.20 < *x* < 0.30 cannot be understood by considering the tolerance factor.

**Figure 13.**
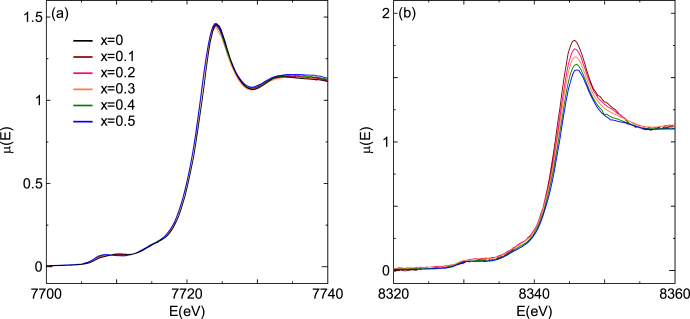
XANES spectra for LaCo_1−*x*_Ni_*x*_O_3±*δ*_ measured at AichiSR. (a) Co K-edges and (b) Ni K-edges.

In addition, the bond valence sum (BVS) for the B-site ions also exhibited an anomaly between 0.20 < *x* < 0.30 and sharply increased for *x* > 0.30. This result is of importance because the BVS should essentially decrease as the Ni^2+^ content increases, i.e. the Co^3+^ content decreases. Considering this anomaly and the ionic radius of Ni (i.e. Ni^2+^ > Ni^3+^), Ni^2+^ and Ni^3+^ are proposed to be dominant for *x* ≤ 0.20 and *x* ≥ 0.30, respectively. Figure 13 shows the x-ray absorption near edge structure (XANES) spectra for LaCo_1−*x*_Ni_*x*_O_3±*δ*_. The Ni K-edge was shifted to the high energy side with the increasing *x*, whereas the Co K-edge did not change with *x*, i.e. the valence of Ni changed from 2+ to 3+ with the increasing *x*, although the valence of Co was independent of the Co/Ni ratio. That is, the valence of Ni is changed by the itinerant carriers in LaCo_1−*x*_Ni_*x*_O_3±*δ*_, whereas the valence of Co is changed by the itinerant carriers in La_1−x_Sr_x_CoO_3±*δ*_. The valence of Ni contributes to the electronic conduction more intensely than the valence of Co in LaCo_1−*x*_Ni_*x*_O_3±*δ*_.

### 6.3. Hall-effect measurements for LaCo_1−x_Ni_x_O_3±*δ*_

**Figure 14.**
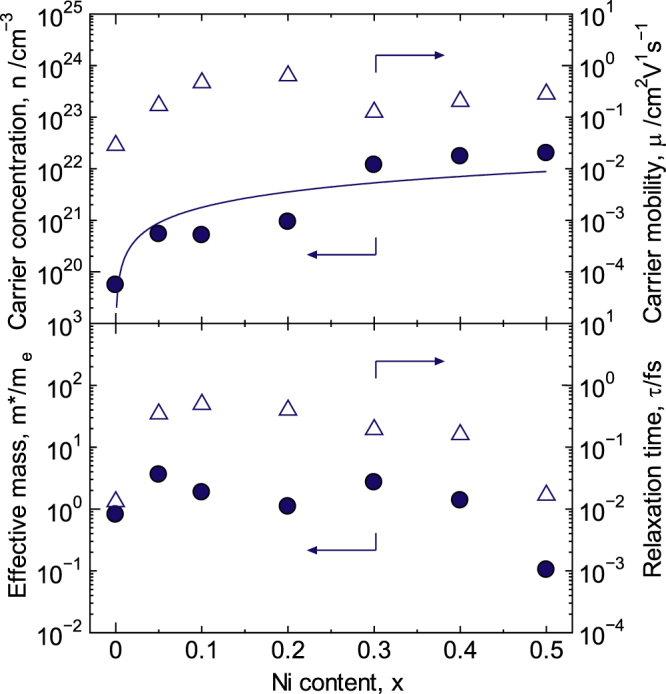
Carrier concentration, *n*, carrier mobility, *μ*, effective mass, *m*∗, and relaxation time, *τ*, for LaCo_1−*x*_Ni_*x*_O_3±*δ*_ at R.T. The solid line indicates the theoretical value of the carrier concentration. Reprinted with permission from [8]. Copyright © 2012 American Chemical Society.

The carrier concentration (*n*) and the mobility (*μ*) were also discontinuous between 0.20 < *x* < 0.30, as shown in figure 14. The Hall coefficient was reversed from negative to positive for 0 < *x* < 0.05, indicating that LaCo_1−*x*_Ni_*x*_O_3±*δ*_ (0.05 ≤ *x* ≤ 0.50) is a p-type conductor and that LaCoO_3_ is an n-type conductor. This p–n transition is caused by the increase in hole concentration by Ni doping to the Co-site according to equation (10). The *n* value increased as *x* increased and reached a maximum of 2.2 × 10^22^ cm^−3^ at *x* = 0.50, which is extremely high for oxides. For 0 ≤ *x* ≤ 0.20, the observed values of *n* were less than the theoretical values*,* which are indicated in figure 14 by the solid line. The oxygen content for *x* = 0.20 was calculated using equations (4) and (10), and the difference in the observed and theoretical values was 2.9310
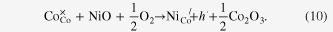



This value is nearly consistent with the value of 2.90 obtained via iodometric titration, indicating that hole generation caused by Ni doping is in a trade-off relation with electron generation (i.e. hole ‘annihilation’) due to the oxygen deficiency for 0 ≤ *x* ≤ 0.20. In contrast, the observed value of *n* exceeded the theoretical value of 0.30 ≤ *x* ≤ 0.50. Therefore, the valence change in Ni with *x* can be concluded to contribute to the *n* value, as mentioned above in this range. On the other hand, the *μ* value reached a maximum of 0.63 cm^2^ Vs^−1^ at *x* = 0.20 and then decreased one order of magnitude for 0.20 < *x* < 0.30. Note that the *μ* value for 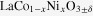 was less than that for La_1−*x*_Sr_*x*_CoO_3±*δ*_ [6, 7, 17].

To analyze the behavior of *μ* in more detail, the DOS effective mass (*m∗*) and the relaxation time (*τ*) were deduced using equations (5)–(8), and the results are shown in figure 14.

Given the absolute values of *m*∗ and *τ*, the difference in the values of *μ* for LaCo_1−*x*_Ni_*x*_O_3±*δ*_ and La_1−*x*_Sr_*x*_CoO_3±*δ*_ can be attributed to *τ*. The small value of *τ* for LaCo_1−*x*_Ni_*x*_O_3±*δ*_ is due to Ni doping to the conduction path of the Co-sites. The values for *m*∗, *n*, *μ*, the B−O bond length and the B−O−B bond angle displayed anomalies between 0.20 < *x* < 0.30. Therefore, the decrease in the *μ* value for 0.20 < *x* < 0.30 is mainly caused by an increase in the *m*∗ value. After the anomalous behavior, *m*∗ decreased as the Ni content increased for 0.30 ≤ *x* ≤ 0.50 and reached 0.10 m_e_ at *x* = 0.50.

**Figure 15.**
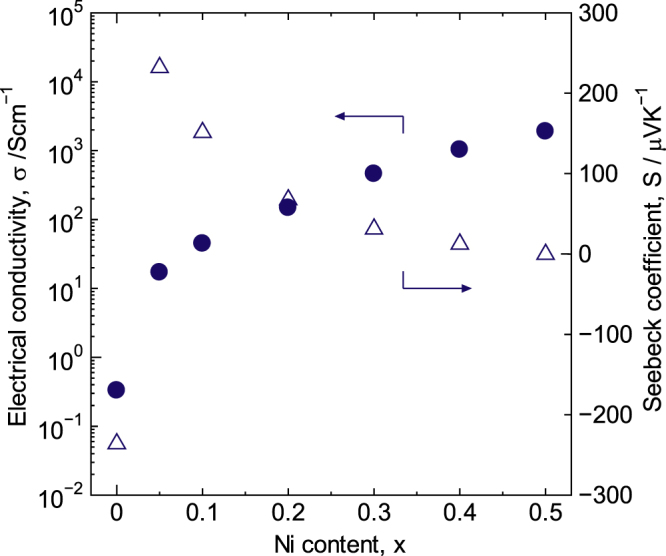
Electrical conductivity, *σ*, and Seebeck coefficient, *S*, for LaCo_1−*x*_Ni_*x*_O_3±*δ*_ at R.T. Reprinted with permission from [8]. Copyright © 2012 American Chemical Society.

The high *σ* value of 1.9 × 10^3^ S cm^−3^ when *x* = 0.50 at R.T. can be attributed to the high *n* value of 2.2 × 10^22^ cm^−3^ and the small *m*∗ value of 0.10 m_e_ for *x* = 0.50. This result is consistent with that observed for La_1−*x*_Sr_*x*_CoO_3±*δ*_. The *σ* and *S* values for LaCo_1−*x*_Ni_*x*_O_3±*δ*_ at R.T. are shown in figure 15. The *σ* value reached a maximum of 1.9 × 10^3^ S cm^−3^ at *x* = 0.50, whereas the *S* value reached a maximum of 230 *μ*V/K at *x* = 0.05. The *S* value gradually decreased as the Ni content increased, approached zero at *x* = 0.50, and then the sign reversed from negative to positive between 0 < *x* < 0.05 as a result of Ni doping. The pure LaCoO_3_ synthesized in this study was n-type, although the sign of LaCoO_3_ remains a matter of discussion, as mentioned above [17, 20, 44]. The behavior of *S* with *x* reflects the increase in *n* and the decrease in *m*∗ with. The increasing *x*.

### 6.4. Band calculations for LaCo_1−x_Ni_x_O_3±*δ*_

The DOS for LaCo_0.5_Ni_0.5_O_3_ was calculated using the hybrid density functional theory employing the full-potential APW+lo method in the WIEN2k code [48–50, 84], as shown in a previous report [8]. Although pure LaCoO3 has a nonmagnetic LS state, the magnetic state wasstabilized in LaCo_0.5_Ni_0.5_O_3_ because of the contribution of both Ni with unpaired electrons and the Co transition to a magnetic IS or HS state due to hole doping. In the up-spin direction, the total density of states (TDOS) did not possess a band gap at *E*_F_, indicating that LaCo_0.5_Ni_0.5_O_3_ is a half metal. A small *m*∗ was expected based on the gradual slope of the TDOS for LaCo_0.5_Ni_0.5_O_3_, although the ground state of pure LaCoO_3_ is a semiconductor with a nonmagnetic state. In addition, Ni 3*d* levels contributed to the valence band more intensely than Co 3*d* levels, suggesting that the itinerant carriers were highly concentrated on Ni, i.e. Ni^3+^ ions were formed according to the Rietveld refinement and XANES analysis, as mentioned above. The down-spin contribution to the conduction was small because the *E*_F_ lies in the band gap. Accordingly, the magnetic state was stabilized by Ni doping, resulting in the small *m*∗. This result is consistent with that for La_0.5_Sr_0.5_CoO_3_, although the dopant is different.

### 6.5. Physical properties of LaCo_1−x_Ni_x_O_3±*δ*_ up to 1173 K in air

**Figure 16.**
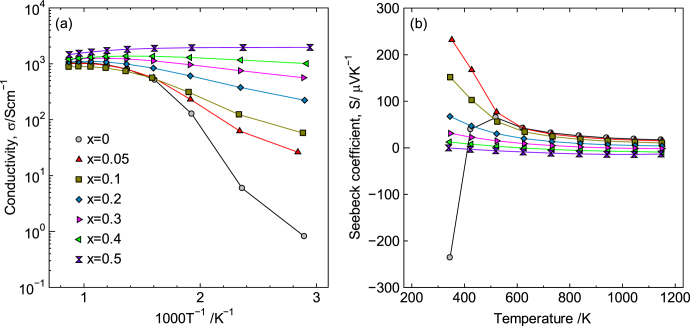
Temperature dependence of (a) electrical conductivity, *σ*, and (b) Seebeck coefficient, *S*, for LaCo_1−*x*_Ni_*x*_O_3±*δ*_. Reprinted with permission from [8]. Copyright © 2012 American Chemical Society.

Figure 16(a) shows the *σ* values for LaCo_1−*x*_Ni_*x*_O_3±*δ*_ samples measured at 373–1173 K in air. The *σ* value for *x* ≤ 0.40 exhibited semiconducting behavior below 800 K but became saturated above 800 K. In contrast, the conductivity for *x* = 0.50 exhibited the largest value and was metallic over the entire temperature range examined, i.e. the *σ* value monotonically decreased with the increasing temperature. In addition, the temperature coefficient of conductivity for *x* = 0.50 was smaller than that of standard metals, indicating that LaCo_1−*x*_Ni_*x*_O_3±*δ*_ for *x* = 0.50 is suitable for use as an oxide electrode and as wiring at high temperatures in air.

Meanwhile, the value for *S* of LaCo_1−*x*_Ni_*x*_O_3±*δ*_ is also interesting. Figure 16(b) shows the temperature dependence of *S*, which decreased and reversed its sign from positive to negative as the Ni content and temperature increased. Between 0.40 ≤ *x* ≤ 0.50, |*S*| remained close to zero, even at high temperatures, because the *m*∗ value was close to zero and the temperature coefficient was small, indicating that the electronic structure was maintained from R.T. to 1173 K. On the other hand, the sign of *S* for pure LaCoO_3_ reversed from positive to negative between 340 K and 420 K. Considering that the sign for nondoped oxides is determined by carriers generated due to nonstoichiometry, as mentioned above, the sign change for pure LaCoO_3_ is believed to be caused by oxygen elimination with the increasing temperature.

The thermogravimetric analysis for LaCo_0.5_Ni_0.5_O_3±*δ*_ up to 1173 K revealed a change of less than 0.05 mass%, i.e. little change in the oxygen content occurred, unlike for La_0.5_Sr_0.5_CoO_3−*δ*_, for which approximately a 2.8 wt% decrease was observed as the temperature was increased to 1173 K. In addition, both the *σ* and *S* values remained unchanged after annealing at 1273 K for 100 h in air. These results indicate that LaCo_0.5_Ni_0.5_O_3±*δ*_ is stable, even at high temperatures in air.

### 6.6. Section summary

The high *σ* value of 1.9 × 10^3^ S cm^−1^ for LaCo_0.5_Ni_0.5_O_3_ is because of a high *n* value of 2.2 × 10^22^ cm^−3^ and a small *m*∗ value of 0.10 m_e_. In addition, the temperature coefficient of *σ* is smaller than that of standard metals, and little change in the oxygen content occurs up to 1173 K in air, indicating that LaCo_0.5_Ni_0.5_O_3_ is stable at high temperatures in air. However, there is no relation between the B–O bond length and *μ*, i.e. *m*∗ in this system, which is unlike La_1−*x*_Sr_*x*_CoO_3±*δ*_ and 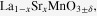 i.e. a decrease in the B–O bond length does not necessarily lead to a decrease in *m*∗ in this system. Considering the results of XAFS analysis and band calculation, we surmise that this behavior is caused by the contribution to the DOS at the Fermi level of Ni 3*d* levels and the change to Ni substitution from Ni doping with the increasing Ni content. Accordingly, the electronic structure, which comprises a flat DOS around *E*_F_ with a magnetic state, is believed to contribute to the small *m*∗ value for these three systems, rather than the crystal structure (B–O bond length).

## 7. Conclusions

The compounds La_1−*x*_Sr_*x*_CoO_3±*δ*_, La_1−*x*_Sr_*x*_MnO_3±*δ*_ and 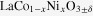 all exhibit high electronic conduction at R.T., with maximum electrical conductivities of 4.4 × 10^3^ S cm^−1^ at *x* = 0.50, 1.5 × 10^3^ S cm^−1^ at *x* = 0.40 and 1.9 × 10^3^ S cm^−1^ at *x* = 0.50, respectively. The high conductivity is because of a high carrier concentration and a small effective mass. The small effective mass is attributed to the flat DOS around the *E*_F_, which is caused by the magnetic state. Of the three systems, LaCo_0.5_Ni_0.5_O_3±*δ*_ is the most suitable for the fabrication of oxide electrodes and wiring because oxide elimination occurs in La_1−*x*_Sr_*x*_CoO_3±*δ*_ as the temperature increases, and the electrical conductivity of La_1−*x*_Sr_*x*_MnO_3±*δ*_ is slightly low at temperatures above 400 K. In LaCo_0.5_Ni_0.5_O_3±*δ*_, the temperature coefficient of the conductivity is smaller than that of standard metals, and little change in the oxygen content occurs up to 1173 K in air. Therefore, we believe that LaCo_0.5_Ni_0.5_O_3_ is suitable as a conductive material for electrodes and electrical wiring that are used at high temperatures in air because it satisfies the requirement properties as oxide electrodes and wiring for ceramic-based products.
